# Dry needling versus platelet-rich plasma in myofascial pain: A randomized trial

**DOI:** 10.6026/973206300212927

**Published:** 2025-08-31

**Authors:** Jyoti Rani, Manisha Lakhanpal Sharma, Siddharth Srivastava, Swati Goel, Yashobanta Biswal, Ashu Rani

**Affiliations:** 1 Department of Oral Medicine and Radiology, ITS Dental College, Hospital and Research Centre, Greater Noida, Uttar Pradesh, India

**Keywords:** MPDS, dry needling, PRP, trigger points, chronic pain

## Abstract

The efficacy of dry needling (DN) and platelet-rich plasma (PRP) in managing trigger points in Myofascial Pain Dysfunction Syndrome (MPDS). Hence, Twenty-two patients were equally divided into DN and PRP groups and evaluated using the Pain Disability Questionnaire (PDQ), Numerical Rating Scale (NRS), maximum mouth opening (MMO) and tenderness at baseline, post-treatment, 4 weeks and 12 weeks. Both groups showed significant improvement in pain and function, but PRP demonstrated superior effectiveness in reducing pain and enhancing jaw mobility at follow-ups. Thus, we show that PRP may be a more effective long-term treatment for MPDS compared to dry needling.

## Background:

Myofascial Pain Dysfunction Syndrome (MPDS) is a prevalent musculoskeletal disorder characterized by the presence of hyperirritable trigger points within taut bands of skeletal muscle. These trigger points elicit localized tenderness, referred pain, muscle stiffness and restricted jaw movements, significantly impairing patients' quality of life. The pathophysiology involves hypoxia, ischemia and altered neuromuscular activity, often exacerbated by factors such as occlusal disturbances, trauma, bruxism, stress and emotional distress [1, 3]. Dry needling is a minimally invasive technique aimed at inactivating myofascial trigger points to reduce pain and improve muscle function. However, current evidence remains inconclusive, with studies showing mixed outcomes and regulatory bodies considering it experimental and investigational [2]. Epidemiologically, MPDS affects 30-93% of the general population, with a higher prevalence among women (3:1 to 5:1 ratio) and individuals aged 20-40 years [3, 6]. Clinical manifestations include jaw dysfunction; TMJ sounds (clicking/popping), headaches and referred pain patterns, necessitating prompt and effective management [3, 4]. Current treatment modalities for MPDS encompass non-invasive approaches (rest, pharmacotherapy, physiotherapy) and invasive interventions (dry needling, platelet-rich plasma (PRP), botulinum toxin injections and surgical options) [5]. Among these, dry needling has emerged as a minimally invasive technique that mechanically disrupts trigger points, alleviating pain and improving mobility with minimal side effects [1, 3]. Conversely, PRP therapy-a novel regenerative approach-utilizes concentrated growth factors to promote tissue healing and reduce inflammation, though its efficacy in MPDS remains understudied [7]. This randomized controlled trial compared the effectiveness of platelet-rich plasma and dry needling in managing masseter muscle trigger points in patients with myofascial pain syndrome. The findings provide clinical insight into minimally invasive approaches for reducing pain and improving muscle function [8]. Despite the high prevalence of MPDS, comparative evidence on the effectiveness of dry needling versus PRP is scarce. Given PRP's potential for tissue regeneration and analgesia, juxtaposed with dry needling's immediate mechanical benefits, this study aims to evaluate and compare their therapeutic outcomes in MPDS patients. Therefore, it is of interest to report the comparative effectiveness of dry needling and PRP in the management of MPDS.

## Methodology:

This randomized controlled trial (RCT), approved by the Atal Bihari Vajpayee Medical University ethics committee and adhering to CONSORT guidelines, compared the efficacy of dry needling (DN) and platelet-rich plasma (PRP) injections for managing trigger points in 22 patients (11 per group) aged 20-40 years with Myofascial Pain Dysfunction Syndrome (MPDS). Patients were randomized into two groups: Group A received DN using acupuncture needles (0.25 x 40 mm) inserted into trigger points to elicit a local twitch response, while Group B received 0.5 mL PRP injections per trigger point, prepared via double centrifugation of 20 mL venous blood. Outcomes assessed at baseline, post-treatment, 4 weeks and 12 weeks, included pain intensity (Numerical Rating Scale), functional impact (Pain Disability Questionnaire), maximum mouth opening (measured in mm) and tenderness (scored 0-10). Statistical analysis using SPSS v21.0 involved non-parametric tests (Mann-Whitney U, Kruskal-Wallis) and chi-square tests, with significance set at p < 0.05. No major adverse events were reported. Despite the small sample size and short-term 12-week follow-up, the study's strengths included its randomized design, standardized protocols and blinded outcome assessment. PRP demonstrated superior long-term efficacy, highlighting its potential as a first-line minimally invasive treatment for MPDS.

## Results:

A randomized clinical trial was conducted with 22 patients diagnosed with Myofascial Pain Dysfunction Syndrome (MPDS), equally divided into two groups: Group A (Dry Needling, n=11) and Group B (Platelet Rich Plasma, n=11). The groups were comparable at baseline in terms of gender distribution (p=0.155), treatment site (p=0.655), age (p=0.972) and number of trigger points (p=0.737), ensuring no confounding variables influenced the outcomes. The study assessed four key parameters-Pain Disability Questionnaire (PDQ), Numerical Rating Scale (NRS), Maximum Mouth Opening (MMO) and Tenderness-at baseline, post-injection, 4 weeks and 12 weeks. Both groups demonstrated improvements across all parameters, but Group B (PRP) consistently showed statistically significant greater reductions in pain, disability and tenderness, as well as greater improvements in mouth opening, compared to Group A (Dry Needling). These findings are summarized in Table 1 (see PDF). This table consolidates the key findings from the thesis, highlighting that both treatments improved clinical outcomes, but PRP (Group B) consistently demonstrated statistically significant greater improvements in pain disability, pain intensity, mouth opening and tenderness compared to Dry Needling (Group A) at post-injection, 4 weeks and 12 weeks.

## Discussion:

This randomized clinical trial compared Dry Needling (DN) and Platelet Rich Plasma (PRP) in Myofascial Pain Dysfunction Syndrome (MPDS). Both DN and PRP improved pain, disability, maximum mouth opening (MMO), and tenderness. PRP showed significantly greater efficacy at post-injection, 4-week and 12-week follow-ups (Table 1 - see PDF). The reduction in NRS and PDQ scoresin the PRP group aligns with earlier studies. Nitecka-Buchta *et al.* (2019) reported 58% pain reduction with PRP versus 10.3% with saline [9]. Sakalys *et al.* (2020) also found PRP reduced pain in masticatory muscles more effectively than lidocaine [10]. The present study showed sustained pain reduction in the PRP group (mean NRS: 0.09) versus DN (mean NRS: 0.73) at 12 weeks. PRP's growth factors promote tissue regeneration and reduce inflammation, providing longer-lasting relief [7, 8]. DN'seffect is attributed to MTrP disruption and modulation of central nervous system excitability [1]. PRP contributes to myogenesis and muscle regeneration, enhancing elasticity and function [7]. DN improved MMO, consistent with Fernandez-Carnero *et al.* (2010) and Garcia-de la-Banda-Garcia *et al.* (2023) [11, 12]. However, PRP showed significantly greater MMO gains at 4 and 12 weeks. This suggests PRP restores muscle length and function more effectively than DN's mechanical action. PRP's sustained efficacy required no repeat injections by 12 weeks. Some DN patients experienced recurrence; necessitating additional sessions. Agarwal *et al.* (2022) also reported PRP outperformed DN in pain reduction and satisfaction [8]. PRP's advantages include autologous origin, reduced infection risk, and simple preparation [11]. Treatment choice may depend on resources, patient preference, and clinician expertise. Study limitations include small sample size (n=22) and short 12-week follow-up. As MPDS is chronic, larger studies with ≥6-month follow-ups are required. Combined DN and PRP therapy was not explored but may enhance outcomes (Nowak *et al.* 2021) [13]. Future research should test larger cohorts, longer follow-up, and combined protocols. Standardizing PRP preparation (e.g., double-spin) and DN needles (0.25 mm) will improve comparability. In conclusion, both DN and PRP are effective for MPDS, but PRP shows superior and sustained outcomes. PRP is promising for long-term relief, while DN remains useful for short-term symptom control.

## Conclusion:

Both Dry Needling and Platelet Rich Plasma (PRP) effectively improve pain, disability, mouth opening and tenderness in Myofascial Pain Dysfunction Syndrome (MPDS), with PRP showing significantly greater and more sustained efficacy across all parameters is shown. PRP's regenerative properties make it a promising treatment, while Dry Needling remains a viable, cost-effective alternative. Larger studies with longer follow-ups are needed to confirm these findings and explore combined therapies.
